# Supplementary material for: PPI-based screening of hub genes related to sepsis migration/pyroptosis and immune infiltration analysis
Source: PLoS One. 2025 Nov 18;20(11):e0336982. doi: 10.1371/journal.pone.0336982 (PMC12626297; doi:10.1371/journal.pone.0336982)
Supplement: S2 Table — (DOCX) [file pone.0336982.s002.docx]

**Table S2** GO analysis of differentially expressed genes related to pyroptosis and migration: Based on the GO database

| ONTOLOGY | ID | Description | p.adjust | qvalue |
| --- | --- | --- | --- | --- |
| BP | GO:0022407 | regulation of ce**l** -cell adhesion | 3.4869 e-13 | 1.2421 e-13 |
| BP | GO:0001819 | positive regulation of cytokine  production | 1.0839 e-12 | 3.8612 e-13 |
| BP | GO:1903037 | regulation of leukocyte ce**l** - cell  adhesion | 1.3718 e-11 | 4.8867 e-12 |
| CC | GO:0045121 | membrane raft | 0.00111589 | 0.00079829 |
| CC | GO:0098857 | membrane microdomain | 0.00111589 | 0.00079829 |
| CC | GO:0098589 | membrane region | 0.00111589 | 0.00079829 |
| MF | GO:0051019 | mitogen-activated protein kinase  binding | 0.00100231 | 0.00048893 |
| MF | GO:0050839 | ce**l** adhesion molecule binding | 0.00335335 | 0.00163578 |
| MF | GO:0030971 | receptor tyrosine kinase binding | 0.0064917 | 0.00316668 |

(GO: Gene Ontology, BP: Biological Process, MF: Molecular Function, CC: Cell Component)
