# Supplementary material for: PPI-based screening of hub genes related to sepsis migration/pyroptosis and immune infiltration analysis
Source: PLoS One. 2025 Nov 18;20(11):e0336982. doi: 10.1371/journal.pone.0336982 (PMC12626297; doi:10.1371/journal.pone.0336982)
Supplement: S3 Table — (DOCX) [file pone.0336982.s003.docx]

Table S3 KEGG analysis of differentially expressed genes related to pyroptosis and migration in cells: based on KEGG database

| ID | Description | p.adjust | qvalue |
| --- | --- | --- | --- |
| hsa04657 | IL-17 signaling pathway | 1.2561 e-07 | 5.4638 e-08 |
| hsa04668 | TNF signaling pathway | 3.8216 e-06 | 1.6623 e-06 |
| hsa04620 | Toll-like receptor signaling pathway | 4.3552 e-05 | 1.8944 e-05 |
| hsa05235 | PD-L1 expression and PD-1  checkpoint pathway in cancer | 0.00030697 | 0.00013353 |
| hsa04621 | NOD-like receptor signaling  pathway | 0.00030697 | 0.00013353 |
| hsa04933 | AGE-RAGE signaling pathway in  diabetic complications | 4.0346 e-05 | 1.7549 e-05 |
| hsa04064 | NF-kappa B signaling pathway | 0.0052342 | 0.00227673 |
| hsa04010 | MAPK signaling pathway | 0.0106807 | 0.0046458 |
| hsa04115 | p53 signaling pathway | 0.02648743 | 0.01152128 |

(KEGG: Kyoto Encyclopedia of Genes and Genomes
