# Supplementary material for: PPI-based screening of hub genes related to sepsis migration/pyroptosis and immune infiltration analysis
Source: PLoS One. 2025 Nov 18;20(11):e0336982. doi: 10.1371/journal.pone.0336982 (PMC12626297; doi:10.1371/journal.pone.0336982)
Supplement: S4 Table — (DOCX) [file pone.0336982.s004.docx]

**Table S4** GSEA analysis of Table4 sepsis and control group: Based on c2.cp.kegg.v7.4.entrez.gmt data set

| Description | NES | p.adjust | qvalues |
| --- | --- | --- | --- |
| WP_PI3KAKT_SIGNALING_PAT  HWAY | 1.45616122 | 0.02267727 | 0.01584774 |
| FISCHER_DIRECT_P53_TARGE  TS_META_ANALYSIS | 1.59133949 | 0.02267727 | 0.01584774 |
| BIOCARTA_CASPASE_PATHWA  Y | 1.8872618 | 0.02267727 | 0.01584774 |
| WP_IL18_SIGNALING_PATHWA  Y | 1.70448613 | 0.02357455 | 0.0164748 |
| REACTOME_SIGNALING_BY_  RHO_GTPASES | 1.78132067 | 0.0248356 | 0.01735607 |
| REACTOME_MAPK6_MAPK4_S  IGNALING | 1.81075749 | 0.02847747 | 0.01990115 |
| KEGG_NOD_LIKE_RECEPTOR_  SIGNALING_PATHWAY | 1.73457501 | 0.04528427 | 0.03164639 |
| MORI_SMALL_PRE_BII_LYMP  HOCYTE_DN | 1.68859292 | 0.04528427 | 0.03164639 |
| REACTOME_TRANSLESION_S YNTHESIS_BY_Y_FAMILY_DN A_POLYMERASES_BYPASSES_  LESIONS_ON_DNA_TEMPLATE | 1.64986958 | 0.04528427 | 0.03164639 |

(GSEA: Gene Set Enrichment Analysis )
