# Supplementary material for: PPI-based screening of hub genes related to sepsis migration/pyroptosis and immune infiltration analysis
Source: PLoS One. 2025 Nov 18;20(11):e0336982. doi: 10.1371/journal.pone.0336982 (PMC12626297; doi:10.1371/journal.pone.0336982)
Supplement: Attachment — Submitted filename: Response to Reviewers.docx [file pone.0336982.s005.docx]

Dear Editor / Editor and Reviewers

**Manuscript ID**: PONE-D-25-35332

**Title**: PPI-based screening of hub genes related to sepsis migration/pyroptosis and immune infiltration analysis

We are deeply grateful to you and the other reviewers for your invaluable comments and suggestions, which have played a crucial role in significantly improving the quality of our manuscript. In response to the thorough and insightful feedback provided, we have conducted extensive revisions to the paper. We have carefully addressed each of the points raised, supplemented relevant content where necessary, and refined several sections to enhance clarity and completeness. A detailed point-by-point response to all the reviewers’ comments has been prepared, and all changes in the manuscript have been highlighted in red for your convenience. We sincerely hope that the revised version meets with your approval and are more than willing to make any further adjustments as needed. Thank you very much for your time and support—it is a great honor to have our work considered for publication in *PLOS ONE*.

1. Please ensure that your manuscript meets PLOS ONE's style requirements, including those for file naming. The PLOS ONE style templates can be found at

RE：We sincerely appreciate your thorough review and valuable comments.

We will meticulously revise the manuscript in strict accordance with the latest *PLOS ONE* template, comprehensively reviewing and adjusting the manuscript structure, formatting, file naming, figure/table uploads, captions, and reference style to ensure all submitted materials fully comply with the journal's requirements.

Thank you once again for your valuable suggestions.

2. PLOS requires an ORCID iD for the corresponding author in Editorial Manager on papers submitted after December 6th, 2016. Please ensure that you have an ORCID iD and that it is validated in Editorial Manager. To do this, go to ‘Update my Information’ (in the upper left-hand corner of the main menu), and click on the Fetch/Validate link next to the ORCID field. This will take you to the ORCID site and allow you to create a new iD or authenticate a pre-existing iD in Editorial Manager.

RE：Thank you for your careful review and valuable comments. We will supplement the ORCID iD of corresponding authors in the author information section in subsequent revisions, and ensure that verification is completed in the Editorial Manager system to meet journal-related requirements.

Thank you again for your valuable advice.

3. Please note that PLOS One has specific guidelines on code sharing for submissions in which author-generated code underpins the findings in the manuscript. In these cases, we expect all author-generated code to be made available without restrictions upon publication of the work. Please review our guidelines at https://journals.plos.org/plosone/s/materials-and-software-sharing#loc-sharing-code and ensure that your code is shared in a way that follows best practice and facilitates reproducibility and reuse.

RE：Thank you for your thorough review and valuable comments. All analytical procedures in this study were performed using R scripts developed by the authors. The relevant code and documentation have been prepared in accordance with the journal’s requirements. Upon publication of the manuscript, all author-generated code and associated documentation will be made publicly available on a mainstream open-source platform. Detailed access information will be provided in the “Data and Code Availability Statement” section of the subsequent manuscript to ensure the reproducibility and reusability of the research findings.

We sincerely appreciate your insightful suggestions.

4. We note that the grant information you provided in the ‘Funding Information’ and ‘Financial Disclosure’ sections do not match.

When you resubmit, please ensure that you provide the correct grant numbers for the awards you received for your study in the ‘Funding Information’ section.

RE：Thank you for your thorough review and valuable feedback. We will carefully verify and standardize the funding information in the subsequent revision to ensure consistency between the "Funding Information" and "Financial Disclosure" sections.

Once again, we appreciate your insightful suggestions.

5. If the reviewer comments include a recommendation to cite specific previously published works, please review and evaluate these publications to determine whether they are relevant and should be cited. There is no requirement to cite these works unless the editor has indicated otherwise.

RE：We sincerely appreciate your thorough review and valuable suggestions. Based on your recommendations, this study will conduct a comprehensive evaluation of the relevance of the suggested literature and will incorporate appropriate citations in subsequent revisions as needed, thereby ensuring the scientific rigor and representativeness of the references cited.

Thank you once again for your insightful comments.

Additional Editor Comments:

Reviewer #1:

Reviewer #2:

[Note: HTML markup is below. Please do not edit.]

Reviewers' comments:

Reviewer's Responses to Questions

Comments to the Author

1. Is the manuscript technically sound, and do the data support the conclusions?

The manuscript must describe a technically sound piece of scientific research with data that supports the conclusions. Experiments must have been conducted rigorously, with appropriate controls, replication, and sample sizes. The conclusions must be drawn appropriately based on the data presented.

Reviewer #1: Yes

Reviewer #2: Yes

2. Has the statistical analysis been performed appropriately and rigorously?

Reviewer #1: Yes

Reviewer #2: Yes

3. Have the authors made all data underlying the findings in their manuscript fully available?

The PLOS Data policy requires authors to make all data underlying the findings described in their manuscript fully available without restriction, with rare exception (please refer to the Data Availability Statement in the manuscript PDF file). The data should be provided as part of the manuscript or its supporting information, or deposited to a public repository. For example, in addition to summary statistics, the data points behind means, medians and variance measures should be available. If there are restrictions on publicly sharing data—e.g. participant privacy or use of data from a third party—those must be specified.

Reviewer #1: Yes

Reviewer #2: Yes

4. Is the manuscript presented in an intelligible fashion and written in standard English?

PLOS ONE does not copyedit accepted manuscripts, so the language in submitted articles must be clear, correct, and unambiguous. Any typographical or grammatical errors should be corrected at revision, so please note any specific errors here.

Reviewer #1: Yes

Reviewer #2: Yes

5. Review Comments to the Author

Please use the space provided to explain your answers to the questions above. You may also include additional comments for the author, including concerns about dual publication, research ethics, or publication ethics. (Please upload your review as an attachment if it exceeds 20,000 characters)

Reviewer #1: 1. Strengths

The bioinformatics pipeline (e.g., batch correction with sva, DEG analysis with limma, enrichment with clusterProfiler) is standard and well-executed, with external validation adding credibility.

Results are logically presented, and the identification of 8 validated hub genes (TLR2, SIRT1, PTGS2, MAPK14, IL18, ICAM1, CD274, CASP3) is supported by ROC curves (AUC >0.7).

Data availability is fully met, and no funding/competing interests are declared.

-Major Concerns and Suggestions

Methods and Results Soundness:

The methods are detailed and reproducible, but some choices (e.g., lenient DEG threshold of logFC >0) could introduce noise. Conclusions are supported, but interpretations occasionally overreach (e.g., miRNA's "critical role" without direct evidence).

RE：We sincerely thank the reviewers for their careful reading and valuable comments. In the current analysis, a relatively lenient threshold of logFC > 0 was adopted, primarily due to the generally limited expression magnitude observed in sepsis blood samples. This threshold was used during the initial screening phase to ensure biological sensitivity. However, in subsequent analyses, multiple layers of filtering and validation using an independent external dataset were applied to enhance the reliability of the results and mitigate potential noise introduced by the initial screening. Regarding the role of miRNAs in sepsis, we will revise the manuscript accordingly to more cautiously emphasize the predicted significance of miRNAs as potential regulatory factors, while explicitly stating that further experimental validation is required to elucidate their specific mechanisms. Detailed modifications can be found in the Discussion section.

Once again, we appreciate your insightful suggestions.

Discussion Section Suggestions:

The Discussion is comprehensive, linking results to sepsis biology and acknowledging limitations (e.g., dataset heterogeneity, lack of wet-lab validation). However, it could be more concise (reduce repetition of intro material) and focused on interpreting results within the study's scope. Strengthen by:

RE：Thank you for your thorough review and valuable comments. We will revise the Discussion section in accordance with your suggestions by reducing redundant background information, placing greater emphasis on the core findings of this study and their scientific significance, and providing a more in-depth interpretation of the relevant signaling pathways and immune mechanisms. Specific revisions are detailed in the Discussion section.

Once again, we sincerely appreciate your insightful recommendations.

Explicitly tying hub genes to sepsis mechanisms without overgeneralizing (e.g., specify that findings are from blood-derived data, limiting tissue-specific inferences).

RE：Thank you for your thorough review and valuable comments. All data analyses and conclusions presented in this study are based on human peripheral blood samples. The mechanistic inferences regarding the relevant hub genes are made solely in response to the molecular characteristics observed in the peripheral blood microenvironment. We will further emphasize the tissue origin of our findings—specifically, the peripheral blood—in key sections such as the abstract, discussion, and conclusion, to avoid extrapolation to other tissues or organs. Additionally, a corresponding limitation will be explicitly addressed in the discussion section.

We once again express our gratitude for your insightful suggestions.

Expanding on implications for bioinformatics in sepsis research, emphasizing how this integrates pyroptosis/migration (supported by your enrichments in IL-17/TNF pathways).

RE：Thank you for your thorough review and valuable comments. In the revised manuscript, we will further incorporate bioinformatics approaches to explore in depth the role of pyroptosis and migration-related mechanisms in sepsis, as well as their regulatory significance within key inflammatory pathways such as IL-17 and TNF. Specific revisions are detailed in the Discussion section.

Once again, we appreciate your insightful suggestions.

Discussing whether the 8 hub genes are novel in the sepsis context or already known in multiple inflammatory settings (e.g., ICAM1 and CD274 are well-established in endothelial activation and immune checkpoint roles across infections and cancers, as per Meng et al., 2016, Genet Mol Res, and Masugi et al., 2017, Gut).

RE：Thank you for your thorough review and valuable comments. In accordance with your suggestions, we have further clarified the existing research background of the eight hub genes in sepsis and other inflammatory conditions in the revised manuscript, supplemented relevant literature, and highlighted the positioning and novelty of this study. Specific revisions can be found in the Discussion section.

Once again, we sincerely appreciate your insightful recommendations.

Clarifying if the study suggests causal mechanisms (e.g., via pathway enrichments) or merely associations (e.g., correlations in immune infiltration), to avoid implying untested causality.

RE：We sincerely thank the reviewers for their thorough evaluation and valuable comments. In both pathway enrichment analysis and immune infiltration analysis, this study was based on statistical correlations and did not make direct inferences regarding causal mechanisms. All results involving pathways and immune cells were described using terms such as “associated” or “enriched,” avoiding any implication of causality. In the Discussion and Conclusion sections, we further explicitly stated that the findings of this study are based on correlational analyses and do not infer causation, while also emphasizing the need for future validation through functional experiments. Relevant revisions have been made accordingly in the Discussion and Conclusion sections.

Once again, we express our gratitude for the insightful suggestions.

Further Analyses to Enhance Robustness (Not Currently Performed):

To bolster the analysis, consider these additions, which are common in bioinformatics studies and would improve confidence in results without requiring new data:

Weighted Gene Co-expression Network Analysis (WGCNA): Identify co-expression modules among the 23 DEGs and correlate with sepsis traits (e.g., severity). This could reveal if hub genes cluster together, strengthening PPI findings (e.g., as in Li et al., 2023, Front Immunol on pyroptosis-related genes).

Single-Cell RNA-Seq Integration: Map bulk DEGs to public scRNA-seq sepsis datasets (e.g., GSE167363) to resolve cell-type specificity (e.g., pyroptosis in neutrophils vs. macrophages).

Survival Analysis: If metadata allows (e.g., from GSE57065), perform Kaplan-Meier on high/low hub gene expression for mortality prediction.

Alternative Cutoff Testing: Perform and report DEG analysis with stricter logFC cutoffs (e.g., |logFC| >0.5 or >1), as low thresholds (|logFC|>0) can inflate gene lists and FDR.

Validation Across Subgroups: Where data allows, repeat analyses on subsets (e.g., adult vs. pediatric, early vs. late sepsis, infection source) to assess robustness of hub gene selection across patient subtypes.

Methodological Comparison: Conduct pathway enrichment with alternative tools (enrichR, g: Profiler) to confirm core findings are not tool-dependent.

Network Analysis Robustness: Re-run hub selection using multiple network centrality metrics (Degree, Closeness, Betweenness) and show overlap with the presented results.

RE：Thank you for your thorough review and valuable feedback. In the current study, we have integrated multiple transcriptomic datasets for comprehensive analysis and validated key findings using external datasets, with a focus on the association between differentially expressed genes, protein–protein interaction networks, and immune infiltration. This approach demonstrates strong systematic and scientific rigor. Due to limitations in sample size, metadata completeness, and clinical information in the available datasets, relevant analyses have not yet been conducted. However, we fully acknowledge the importance of such analyses for in-depth mechanistic studies. In the future, we plan to incorporate more extensive clinical samples and multi-omics data to further expand related analyses, thereby continuously enhancing the breadth and depth of this research.

Once again, we appreciate your insightful suggestions.

Sensitivity analyses test result stability under varied parameters. Suggested ones:

DEG Threshold Variation: Re-run with logFC >0.5 or >1 to assess if the 23 DEGs/hubs change (common to check robustness, e.g., in Gao et al., 2024, Gene).

RE：Thank you for your thorough review and valuable feedback. In the analytical process of this study, a lenient threshold of logFC > 0 was adopted during the initial screening phase, primarily due to the objective characteristic of generally low expression levels of genes in sepsis blood samples. The rationale behind this setting was to maximize the retention of potential biological signals, ensure biological sensitivity in the preliminary screening results, and avoid premature filtering of key differentially expressed information due to overly stringent thresholds. However, through multi-level rigorous filtering and validation using an independent external dataset in subsequent analyses, the potential influence of non-specific signals from the initial screening phase on the final conclusions was effectively mitigated.

We appreciate your insightful suggestions once again.

Bootstrap Resampling for Enrichments: Bootstrap GO/KEGG (1000 iterations) to compute confidence intervals for p-values, reducing false positives from small gene sets.

RE：Thank you for your thorough review and valuable feedback. In the GO/KEGG enrichment analysis, this study employed the standard workflow of the clusterProfiler package, incorporating multiple hypothesis testing (Benjamini–Hochberg correction) to strictly control the false discovery rate. This analytical pipeline has been widely adopted in functional enrichment studies and is well-suited to the sample size and gene set scale of the present research, thereby adequately supporting the validity and reliability of the core findings. We highly value the suggestion regarding further enhancing analytical robustness through methods such as Bootstrap resampling. However, given the number of gene sets and the analytical depth in this study, the current statistical strategy is considered sufficient to meet the research objectives. Should future investigations involving smaller gene sets or specific functional pathways require enhanced statistical robustness, we will actively consider incorporating the Bootstrap approach and provide corresponding details in the Methods and Discussion sections.

We appreciate your insightful comments once again.

PPI Interaction Score Variation: Test STRING scores >0.7 (high confidence) vs. 0.4 to evaluate hub stability.

RE：Thank you for your thorough review and valuable feedback. In this study, a medium confidence threshold (score > 0.4) was adopted to balance the reliability of interaction data with network coverage, aiming to systematically explore potential biological mechanisms. We acknowledge that increasing the threshold (e.g., score > 0.7) could enhance the strength of interaction evidence; however, it may also lead to the exclusion of certain important yet understudied proteins, thereby affecting network connectivity and the comprehensiveness of subsequent analyses. Our current analytical pipeline integrates multiple centrality algorithms and module analysis methods to ensure the stability and reproducibility of hub gene identification. We greatly appreciate your suggestion, and in future studies, if further analyses are required, we will consider performing supplementary analyses under higher confidence thresholds to further evaluate the robustness of hub gene selection.

Thank you once again for your insightful comments.

Cross-Validation in ssGSEA: Split data into training/test sets for immune correlations to avoid overestimation.

RE：Thank you for your thorough review and valuable feedback. In this study, the ssGSEA-based immune infiltration correlation analysis was primarily employed to explore potential associations between the gene and the immune microenvironment, and its analytical procedure is consistent with the mainstream practices in the field. The purpose of correlation analysis is to identify overall trends in the data and generate hypotheses for further in-depth investigation. In our research, key findings were analyzed and externally validated using independent datasets to enhance the robustness and reference value of the results. Regarding strategies such as cross-validation and training/test set partitioning, these approaches can indeed further improve the rigor and generalizability of correlation analyses. In future studies, we plan to incorporate larger sample sizes and multi-center data to further optimize and refine immune correlation analysis methods, giving full consideration to strategies such as cross-validation to mitigate potential overestimation of correlations.

Once again, we appreciate your insightful suggestions.

Partial Rank Correlation or Variance-Based Sensitivity Analyses: Apply in gene/pathway prioritization steps to check for consistent ranking under parameter perturbations.

RE：Thank you for your thorough review and valuable feedback. In this study, multiple mainstream network analysis algorithms were employed for cross-validation during gene and pathway prioritization, ensuring the scientific soundness and rationality of the screening results. Regarding your suggestion to further incorporate sensitivity analysis in the prioritization step to evaluate the robustness of the findings, we take it very seriously. Given that the current analytical framework is primarily based on statistical significance and network properties, and does not yet include sensitivity analysis methods related to parametric modeling, we plan to consider supplementing the evaluation with consistency assessment under parameter perturbation conditions in future work. This will allow for quantitative testing of the stability of core gene ranking results, thereby enhancing the reliability and biological interpretability of our findings.

Once again, we sincerely appreciate your insightful suggestions.

How were quality control (QC) checks performed before and after normalization? Were outlier samples removed?

RE：Thank you for your thorough review and valuable feedback. During the data preprocessing stage, we evaluated the expression distribution and clustering of samples using box plots and principal component analysis (PCA) both before and after normalization and batch effect correction, as detailed in the Results section of the manuscript (Fig. 2A–2D). Throughout the quality control process, no significant anomalies or outlier samples were detected; therefore, all samples were retained for subsequent analyses. All included samples underwent the aforementioned multiple quality assessments, ensuring the scientific validity and reliability of the data analysis.

We appreciate your insightful suggestions once again.

Were PCA plots quantitatively assessed (variance explained by PC1/PC2 before vs. after correction) rather than visually?

RE：Thank you for your thorough review and valuable comments. In this study, the evaluation of batch effect correction was primarily conducted using PCA visualization and box plots, which effectively illustrate data distribution and the outcomes of batch correction, aligning with standard bioinformatics workflows. As data preprocessing is not the central focus of this research, additional quantitative metrics such as the proportion of variance explained by principal components were not included. We highly value your suggestions and will further refine related analyses in subsequent studies.

Thank you once again for your insightful recommendations.

Was log2 transformation applied consistently?

RE：Thank you for your thorough review and valuable comments. In the data preprocessing phase of this study, the raw expression data were uniformly subjected to log₂ transformation, and subsequent analyses were conducted following normalization and batch effect correction to ensure data scale consistency and the reliability of analytical outcomes. We have further supplemented and clarified the relevant methodological descriptions in the revised manuscript.

Once again, we appreciate your insightful suggestions.

Which immune cell signature set was used for ssGSEA—LM22 (CIBERSORT) or another? Was it validated for whole blood transcriptomics?

RE：Thank you for your thorough review and valuable comments. The ssGSEA analysis in this study utilized the 28 human immune cell subtype marker gene sets proposed by Bindea et al. (Immunity, 2013). These gene sets have been widely applied and validated in numerous transcriptomic studies involving whole blood and peripheral blood, and are therefore suitable for the whole blood expression profile data used in this research.

Once again, we appreciate your insightful suggestions.

Were multiple testing corrections applied to the gene–cell correlation analyses?

RE：Thank you for your thorough review and valuable comments. In this study, the analysis of gene–immune cell correlations was performed using ssGSEA and Pearson methods, with the primary objective of conducting an initial screening of a large number of gene–cell correlations to identify candidate gene–cell pairs with potential biological significance, thereby providing a basis for subsequent in-depth functional studies and experimental validation. Therefore, multiple testing correction was not applied to the correlation analysis results at this stage. We acknowledge the importance of multiple testing correction in large-scale correlation analyses for controlling false positives. In future work, we will refine the relevant statistical procedures and, in conjunction with larger sample sizes and functional experiments, apply more stringent statistical corrections and biological validation to the preliminary screening results.

We thank you again for your valuable suggestions.

Could strong positive/negative correlations be due to overall neutrophil or lymphocyte proportions rather than functional associations?

RE：Thank you for your thorough review and valuable comments. In the immune infiltration analysis of this study, the ssGSEA method was employed to infer the relative abundance of various immune cell types. As you rightly pointed out, the strong positive/negative correlations observed between genes and immune cells may be influenced by overall changes in immune cell composition, rather than solely reflecting functional regulation. Therefore, when interpreting the correlation results, we emphasized the need to cautiously understand these associations within the context of immune cell distribution.

Once again, we express our gratitude for your insightful suggestions.

Reviewer #2: Comments to the Author

I appreciate the opportunity to review this manuscript, which addresses a highly relevant clinical issue: the identification of key genes related to sepsis, cell migration, and pyroptosis through bioinformatics analysis. The study is well-structured and represents a potentially valuable contribution to the understanding of sepsis pathophysiology. Below I provide my observations.

Strengths

Clear and specific title, focused on the central aim of the study.

The introduction adequately contextualizes the clinical relevance of sepsis and the need for new biomarkers.

The importance of miRNAs, cell migration, and pyroptosis in the pathogenesis of sepsis is well justified.

The bioinformatics methodology is robust, integrating multiple datasets and employing enrichment analyses, PPI networks, and external validation.

Results are presented in a logical and progressive manner:

Identification of 3,566 DEGs.

Selection of 23 genes related to pyroptosis/migration.

Validation of 8 key genes (TLR2, SIRT1, PTGS2, MAPK14, IL18, ICAM1, CD274, and CASP3).

Relevant biological pathways (IL-17, TNF, TLR) are identified, and immune infiltration analysis highlights meaningful correlations.

The conclusion appropriately proposes that the validated genes may represent potential biomarkers and therapeutic targets.

Overall, the manuscript is clearly written, requiring only minor stylistic and grammatical adjustments for international publication.

Areas for Improvement

The main weakness is the lack of experimental validation (in vitro or in vivo), which limits the clinical translation of the findings. It is recommended to emphasize that results should be considered exploratory and require confirmation in experimental models.

RE：We sincerely appreciate your thorough review and valuable comments. This study, based on large-sample, multi-center, high-throughput data and systematic bioinformatics analyses, provides a theoretical foundation for exploratory research into the molecular mechanisms of sepsis. Due to current constraints in experimental conditions and research timelines, in vitro and in vivo experiments have not yet been conducted concurrently. However, we highly value the relevant suggestions and will further emphasize the exploratory nature of our findings and the necessity for subsequent experimental validation in the revised manuscript. Specific revisions have been made in the Discussion section.

Thank you once again for your insightful recommendations.

There is heterogeneity across datasets (different platforms and patient characteristics). Although batch correction was applied, a more detailed discussion of the potential impact of these differences would strengthen the manuscript.

RE：Thank you for your thorough review and valuable feedback. In the revised manuscript, we will further elaborate on the potential impact of dataset heterogeneity, including a discussion of the technical and biological variations arising from differences in platforms and patient characteristics. We will also supplement relevant robustness validation measures and outline future research directions. Specific revisions can be found in the Materials and Discussion sections of the article.

Once again, we appreciate your insightful suggestions.

The discussion could be enhanced in two key aspects: Clinical applications, contrasting the proposed genes with currently used biomarkers (e.g., procalcitonin, CRP).

RE：Thank you for your thorough review and valuable feedback. Regarding clinical applications and comparisons with existing biomarkers (such as PCT and CRP), the manuscript has already addressed the use of traditional biomarkers in the diagnosis and treatment of sepsis in the Introduction, Discussion, and Conclusion sections, while briefly noting their limitations in sensitivity and specificity. We further elaborated on the association between the key genes identified in this study and the pathophysiology of sepsis, as well as their potential advantages in molecular subtyping and prognostic evaluation. As this study is based on bioinformatics data mining and integrative analysis, a direct comparison or joint validation with traditional clinical biomarkers has not yet been conducted. In subsequent research, we plan to systematically evaluate the complementary value of novel genetic markers and traditional biomarkers such as PCT and CRP in diagnosis and prognosis assessment using clinical samples, with the aim of providing stronger evidence for clinical practice.

We once again express our gratitude for your insightful suggestions.

The relationship with secondary complications of sepsis, such as cardiac dysfunction, renal injury, or lung injury.

RE：We sincerely appreciate your thorough review and valuable comments. In the Discussion section, this study systematically untangles the association between the identified key genes and sepsis-induced subsequent multi-organ injury. By integrating previous research findings, we provide a detailed explanation of the mechanistic roles of molecules such as TLR2, SIRT1, MAPK14, IL-18, and PTGS2 in the initiation and progression of inflammatory responses and organ dysfunction, further revealing the potential regulatory networks and clinical implications of these genes in multi-organ injury. In the revised manuscript, the Discussion section has been expanded with reference to current literature to elaborate on the roles of the aforementioned genes in sepsis-related damage to organs such as the heart, kidneys, and lungs.

Once again, we thank you for your insightful suggestions.

Including a perspective on epigenetic processes (particularly DNA methylation) would enrich the analysis, as these mechanisms regulate gene expression and may affect the identified genes.

RE：Thank you for your thorough review and valuable comments. The current study focuses on systematic bioinformatics analysis at the transcriptome level, primarily investigating sepsis-related genes and their regulatory networks based on expression profile data, and has not yet included mechanistic analyses at the epigenetic level (such as DNA methylation, etc.). We fully acknowledge the important role of epigenetic regulation—particularly DNA methylation and related mechanisms—in the regulation of gene expression and in the pathogenesis and progression of diseases. We plan to actively expand our research in this direction in subsequent studies, thereby further enriching the discussion on the genetic and epigenetic regulatory mechanisms of sepsis.

Once again, we appreciate your insightful suggestions.

Relevant proteins involved in cell migration, such as matrix metalloproteinases (MMPs), were not considered. A brief discussion of their role would complement the analysis.

RE：Thank you for your thorough review and valuable feedback. Matrix metalloproteinases (MMPs) play a dual regulatory role in the pathogenesis of sepsis: in the early inflammatory phase, they promote the recruitment of inflammatory cells and exacerbate tissue injury, whereas in the later phase, they contribute to tissue healing and remodeling. Abnormal upregulation of MMP activity is closely associated with sepsis-related organ damage, suggesting their potential clinical value as therapeutic targets. In our research pipeline, MMP family members such as MMP2 and MMP9 were initially included in the cell migration-related gene set. However, during subsequent differential expression screening and enrichment analysis, some MMPs were excluded from the final core gene network due to screening thresholds and expression variability. Therefore, significant enrichment or identification of MMPs as hub genes was not observed in the current results.

We appreciate your insightful comments once again.

Some network figures (PPI, KEGG, mRNA-miRNA) are complex and difficult to interpret; highlighting key genes more clearly would improve readability.

RE：Thank you for your thorough review and valuable comments. In the construction and presentation of the network graph, this study emphasizes a comprehensive depiction of the interactions and regulatory relationships among differentially expressed genes. Core genes identified through algorithmic screening have been annotated in the figure legend and text descriptions to facilitate readers' systematic understanding of the overall network structure and research focus. Given that the study objective prioritizes holistic network analysis, the current format of graphical presentation better aligns with the analytical requirements.

We appreciate your insightful suggestions once again.

The manuscript could benefit from reducing redundancies in the text (e.g., repeated gene lists in multiple sections) to improve conciseness.

RE：We sincerely thank the reviewers for their careful evaluation and valuable comments. During the revision process, we will further refine and optimize the relevant content, reduce redundancy such as repeated gene lists in the main text, and improve the conciseness and logical flow of the presentation. Specific revisions have been incorporated throughout the manuscript.

Once again, we express our gratitude for your insightful suggestions.

Overall Recommendation

The manuscript presents a solid and well-structured bioinformatics analysis that identifies genes of interest in sepsis. However, to strengthen its scientific impact, I recommend:

Expanding the clinical and comparative discussion.

Emphasizing methodological limitations more explicitly.

Refining text, figures, and references.

RE：We sincerely thank the reviewers for their thorough evaluation and valuable comments. In response to the suggestions provided, we will further refine the clinical and comparative discussions in the Discussion section. In the Methods section, we have provided more explicit descriptions of platform heterogeneity, sample representativeness, and relevant influencing factors in the bioinformatic analyses. The scope and limitations of the statistical analyses and subsequent experimental validations have also been further clarified. The entire manuscript has undergone meticulous revision in terms of expression and reference formatting to enhance its scientific rigor and readability. Specific revisions can be found in the Discussion section of the article.

Once again, we express our gratitude for the insightful recommendations.

With these improvements, the article could provide a significant contribution and serve as a solid foundation for future translational studies in sepsis
